# Hypertonic Solution in Severe COVID-19 Patient: A Potential Adjuvant Therapy

**DOI:** 10.3389/fmed.2022.917008

**Published:** 2022-06-21

**Authors:** Matheus Gennari-Felipe, Leandro Borges, Alexandre Dermargos, Eleine Weimann, Rui Curi, Tania Cristina Pithon-Curi, Elaine Hatanaka

**Affiliations:** ^1^Instituto de Ciências da Atividade Física e Esportes, Universidade Cruzeiro do Sul, São Paulo, Brazil; ^2^Seção de Produção de Imunobiológicos, Centro Bioindustrial, Instituto Butantan, São Paulo, Brazil

**Keywords:** coronavirus, leukocytes, neutrophils, immune system, NETs, NETosis, NaCl

## Abstract

Coronavirus disease 2019 (COVID-19) features hyper-inflammation, cytokine storm, neutrophil function changes, and sodium chloride (NaCl) homeostasis disruption, while the treatment with NaCl hypertonic solutions (HS) controls electrolytic body homeostasis and cell functions. HS treatment is a simple, popular, economic, and feasible therapy to regulate leukocyte function with a robust anti-inflammatory effect in many inflammatory diseases. The purpose of this narrative review is to highlight the knowledge on the use of HS approaches against viral infection over the past years and to describe the mechanisms involved in the release of neutrophil extracellular traps (NETs) and production of cytokine in severe lung diseases, such as COVID-19. We reported the consequences of hyponatremia in COVID-19 patients, and the immunomodulatory effects of HS, either *in vitro* or *in vivo*. We also described the relationship between electrolyte disturbances and COVID-19 infection. Although there is still a lack of clinical trials, hypertonic NaCl solutions have marked effects on neutrophil function and NETs formation, emerging as a promising adjuvant therapy in COVID-19.

## Introduction

The World Health Organization (WHO) determined the severe acute respiratory syndrome coronavirus 2 (SARS-CoV-2) disease (COVID-19) as a pandemic in March 2020. Even after efficient vaccines are made available, the new variants of SARS-CoV-2 may reduce their effectiveness, and COVID-19 caused by different variants can still affect the respiratory tract (1).

COVID-19 patients have also been reported to have superinfections and coinfections with SARS-CoV-2 (2) and a recent meta-analysis found coinfections (19%) and superinfections (24%) of these patients, both being related to the risk of higher mortality (3). In this sense, superinfections and respiratory coinfections in SARS-CoV-2-positive patients were more prevalent in critically ill COVID-19 patients (4) and Paolucci et al. found a correlation between elevated viral load (especially Epstein-Barr virus) and lymphopenia, which demonstrates the relationship between viral prevalence and immunosuppression (5). Thus, alternative practices are needed to prevent hyper-inflammation and the respective worsening of severe respiratory illness in COVID-19 patients.

The vaccine minimizes the risk of coronavirus infections and reduces the severity of the disease, while the heterologous and homologous sera treatments block the virus. In general, antivirals block the activity of a specific enzyme/protein needed for the virus to replicate. In the chronology of disease, vaccine administration must be before contamination, thus, treatment with serums is effective in the high viral load.

The evolution of COVID-19 to lung inflammation results in severe cases. At pulmonary complications, anti-inflammatory drugs (cortisone/dexamethasone) are administrated to patients and, theoretically, in a critical inflammatory period, hypertonic saline could be a potential adjuvant therapy. Hypertonic sodium chloride solution (HS) has hemodynamic and electrolyte homeostasis maintenance properties. It improves blood viscosity, causes fast distension of intravascular volume, and decreases endothelial and tissue edema (6, 7). HS also regulates leukocyte function and exhibits an anti-inflammatory effect to improve inflammatory disease conditions such as sepsis and cystic fibrosis (8, 9).

Sputum induction by HS inhalation has been suggested for use in chronic obstructive pulmonary disease (10), while HS inhalation enhances the effectiveness of respiratory physiotherapy in patients with bronchiectasis (11). HS also stimulates mucociliary clearance in healthy individuals and patients with asthma, bronchiectasis, and cystic fibrosis (12, 13).

Recent studies reported that hyponatremia is associated with poor outcomes in COVID-19 patients as an independent predictor of in-hospital mortality (14, 15). Kimura et al. studied outpatients with COVID-19 without acute respiratory distress syndrome, one of the leading causes of mortality in COVID-19 patients, and suggested considerable symptom resolution with HS administration (16). However, they only enrolled patients who could self-isolate and perform irrigation in a bathroom separate from other household contacts and similar precautions would need to be taken by any COVID-19 patient considering this type of intervention in other studies. It is also worth noting that their study had a reduced number of participants (only 14 participants in the hypertonic saline group; 16). Therefore, despite the positive HS effects in other inflammatory diseases (17, 18), it has not been used in COVID-19 patients, and the mechanisms by which HS regulates mucociliary clearance and neutrophil functions remain unknown. We explored how HS modulates leukocyte function and attenuates severe respiratory illness aggravation in COVID-19 patients. We also discuss how HS may regulate neutrophil functions and NETosis formation in the interaction with SARS-CoV-2.

## Discussion

### Hyponatremia in Coronavirus Diseases 2019 Patients and Hypertonic Solution Administration Approaches

The infection of human cells by SARS-CoV-2 occurs through virus Spike protein binding to angiotensin I-converting enzyme 2 (ACE2) receptor (19). ACE-2 is one of the major anti-regulatory proteins of the main axis of the renin-angiotensin system (RAS), a fundamental determinant for the regulation of electrolyte balance and blood pressure. The binding of SARS-CoV-2 to ACE2 results in increased angiotensin II activity, as well as inflammation, and reduces the counter-response of ACE2 on RAS, which influences electrolyte control and elevates blood pressure (20). Moreover, approximately 60% of COVID-19 patients with watery diarrhea have moderate hyponatremia (21). As a result, SARS-CoV-2 infection promotes disturbances in the homeostasis of pH and electrolytes *in vivo*.

Hyponatremia is the most common electrolyte disturbance and, even when mild, is related to higher mortality (22). This electrolyte disorder is as high as 30% in inpatient settings (23). It is categorized in euvolemic, hypovolemic, and hypervolemic hyponatremia, each managed differently (24). Regarding severe acute respiratory syndrome (SARS), retrospective research in 77 medical, surgical, and mixed intensive care units showed that hyponatremia is described as an independent predictive indicator of poor outcomes in critically ill patients (25) and it is associated with poor prognosis (26). In addition to the impact on lung function, the clinical evolution of patients with COVID-19 can be unpredictable, leading to systemic complications and affecting different organs, as shown in Supplementary Table 1.

Direct endothelial cell infection by SARS-CoV-2 and the following endothelitis can induce platelet activation, high vascular permeability, raised thrombin generation, and reduced fibrinolysis, leading to a hypercoagulable state (27). In severe COVID-19, the high concentration of cytokines is related to systemic and local endothelial dysfunction and injury (28). In turn, endothelial activation induces downregulation of thrombomodulin expression, elevated tissue factor expression, and loss of heparin sulfate – all defensive mechanisms against thrombosis (29), contributing substantially to mortality and morbidity (30). Moreover, unregulated inflammatory markers production can induce blood-brain-barrier disruption, hence favoring the entry of cytokines, (or even SARS-CoV-2) into the central nervous system (31), promoting neurologic damage through either the complement or macrophages (32).

Extra-pulmonary manifestations such as gastrointestinal (GI) symptoms are also common in patients with COVID-19. Since ACE2 is expressed by various tissues, including epithelial cells of the GI tract (33), GI symptoms are usual in COVID-19 and the most ordinary GI presentation in COVID-19 patients is diarrhea, followed by vomiting and/or nausea and abdominal pain (34). Other usual GI symptoms reported in COVID-19 patients are anosmia, anorexia, and dysgeusia (35).

Since SARS-CoV-2 moves into the mucous membranes, it can access the biliary system through the portal vein. Thus, SARS-CoV-2 can induce direct immune injury to hepatocytes (cytopathic effect). In this sense, direct viral cytopathy with micro-vesicular steatosis, mild lobular, or portal implication has been observed in other studies (36). Elements that may influence the hepatic involvement in COVID-19 include exacerbated immune responses/systemic inflammatory response syndrome (37), direct viral cytopathic effects, hypoxia-induced alterations, endothelitis (38), vascular changes due to coagulopathy, and drug-induced liver injury (37). Regarding the severity, the prevalence of liver damage in severe COVID-19 cases (74.4%) was higher than that of patients with mild disease (43%), while the prevalence of liver injury in COVID-19-related deaths was 58% (39).

The etiology of hyponatremia seems to have multiple causes in COVID-19 patients, possibly including the syndrome of inappropriate secretion of antidiuretic hormone (SIADH) and digestive losses of sodium by vomiting or diarrhea (40). COVID-19 aggravation has been related to the reduction in potassium, calcium, and sodium serum levels (41, 42). Berni et al. (43) reported that hyponatremia is related to a more severe outcome in COVID-19 patients (43).

Hyponatremia may also be a consequence of the increased inflammatory biomarkers and interleukin (IL)-6 being one of the most relevant cytokines involved in COVID-19 infection (43). IL-6 may induce hyponatremia by causing vasopressin release (44). Besides, the proposed mechanisms for SIADH in COVID-19 patients involve inflammatory cytokine production (45). This issue requires prospective multicenter research and search for chronic underlying hyponatremia and evaluation of cytokines associated with urinary osmolarity to find out the mechanisms involved in the SIADH and COVID-19 linkage.

*In vitro* assays show that intracellular energy deprivation and membrane depolarization are potential pathways by which the HS usefully avoids virus replication. This possibility was recently raised by Machado et al., who studied non-human primate kidney cell line Vero and found that 1.2% NaCl restrained virus replication by 90%, achieving 100% restraining at mildly HS (1.5%). They also found that 1.1% NaCl was enough to restrain virus replication by 88% in human epithelial lung cell line Calu-3 (46).

In addition to the possible protection against viral replication, several NaCl solutions promote clear patient clinical improvements. HS improves the efficacy of respiratory physiotherapy in patients with bronchiectasis or cystic fibrosis (47, 48), stimulating cough (49), and restraining epithelial sodium channels (6).

Ho et al. investigated a case of SARS-CoV-2 induced SIADH manifesting as new-onset seizures using a pro-active desmopressin strategy (3% HS infusion with concomitant fluid restriction). On day 4, the authors found a clinical recovery with resolution and normalization of natremia. They suggested that high cytokine concentrations promote osmoregulation impairment, leading to hyponatremia (50). Nevertheless, this research is a case study without a control group and based on just one patient’s clinical and biochemical data. A randomized controlled trial (RCT) also reported the efficiency of HS (3, 2.5, 2.0, and 1.5%; nasal irrigation and gargle versus standard care) as therapy on adults within 48 h of the upper respiratory tract infection (URTI) onset. The authors found a reduction in the time of illness, transmission within household contacts, over-the-counter medications use, and viral shedding (51). Accordingly, the analysis from the Edinburgh and Lothians Viral Intervention Study RCT demonstrated that HS (gargling and nasal irrigation) decreases the time of URTI by an average of two-and-a-half days (52), however, since the results are a *post hoc* secondary analysis of data from a pilot RCT, they need to be interpreted with caution. In a systematic review, Singh et al. pointed out that HS with gargles and nasal wash may be beneficial in the prevention and care of COVID-19 patients (53).

### Hypertonic Saline as an Immunomodulatory Agent

During SARS-CoV-2 infection, hyper-inflammation and pulmonary edema are the most worrying clinical conditions (54). COVID-19 in the severe phase presents a cytokine storm with increased plasma concentrations of tumor necrosis factor (TNF)-α, IL-1β, IL-2, IL-6, IL-7, IL-8, IL-10, IL-17, chemokine ligand 2 (CCL2), chemokine C-C motif ligand 3 (CCL3), granulocyte colony-stimulating factor (G-CSF), interferon (IFN)γ, and IFNγ-inducible protein 10 (55, 56). High plasma concentrations of cytokines and chemokines in patients with COVID-19 are associated with the aggravated state of the disease compared to non-severe patients (57). Huang et al. reported that COVID-19 patients in the intensive care unit, compared with non-intensive care unit patients, have increased plasma levels of CCL2, CCL3, interferon-inducible protein 10, TNF-α, IL-2, IL-7, IL-10, and G-CSF (58). Patients with COVID-19 and hyponatremia have a worse prognosis than individuals without electrolyte disbalance (59).

The reduction in cytokine production by leukocytes may be a supplementary beneficial effect of HS on the exacerbated immune response in COVID-19 patients. The HS modulates the expression and release of adhesion molecules, such as beta2-integrins and intercellular adhesion molecule (ICAM)-1, and cytokines (e.g., TNF and IL-10) in leukocytes (60–62).

Aerosolized HS elevates IL-8 release by cystic fibrosis gland cells *via* nuclear factor (NF)-κB pathway (63) and IL-8 expression *via* p38 mitogen-activated protein kinases in human bronchial epithelial cells (64). On a cellular level, the favorable outcomes of aerosolized HS were also reported regarding suppressing mTOR activity in mononuclear cells (65) and the decreased arachidonic acid leukotriene-B4-induced priming of the respiratory burst in neutrophils (66). Oreopoulos et al. reported that HS decreases TNF and increases IL-10 stimulated by lipopolysaccharide (LPS) at the gene expression level, independent of NF-κB signaling. HS treatment might exert its effects by independently modulating pro- and anti-inflammatory molecules, explaining the reduced degree of injury in multiple organs after HS administration (62).

The potential anti-inflammatory effects of HS are still unconcluded in humans. Paff et al. carried out a double-blind RCT study on the impact of HS inhalation (7%, twice daily) in 22 patients with primary ciliary dyskinesia. The authors evaluated inflammatory parameters [serum C-reactive protein, erythrocyte sedimentation rate, white blood cell count and cell differentiation, sputum cell differentiation, sputum neutrophil elastase (NE), IL-1β, IL-6, IL-8, IL-10, TNF-α, myeloperoxidase, IFN-α, and –β] and the quality of life (QoL) of the patients. The authors reported that the QoL-bronchiectasis health perception scale improved with HS. However, there was no alteration in the inflammatory measurements even after 12 weeks of treatment (67). Similarly, Aitken et al. did not find a reduction in IL-8 levels after sputum induction (3% HS at 5-time points over 20 min) in 10 clinically stable patients with cystic fibrosis (68). Elkins et al. also did not find differences in pro-inflammatory cytokines (IL-10, IL-6, IL-8, and TNFα) in the sputum of 164 patients with stable cystic fibrosis (7% HS inhaled twice daily) after 48 weeks of intervention (69). Nevertheless, it is essential to mention that all samples from Aitken et al. and Elkins et al. studies were in the post nebulization condition. There was no direct comparison between participants pre and post-nebulization (68, 69). In contrast, Reeves et al. found that HS reduces neutrophil chemotaxis and IL-8 levels in the sputum of 18 cystic fibrosis patients (nebulized 7% HS) compared to 14 non-cystic fibrosis control subjects, thereby assisting resolution of inflammation in the lower airways (18).

Consensus opinions of experts in hyponatremia by the Hyponatremia Treatment Guidelines (24) indicate that the treatment of hyponatremia depends on two factors: (a) etiology and (b) the volume status and comorbidities of the patient. Besides, usual saline therapies involve two different approaches: fluid restriction treatment and electrolytic substitution treatment. In the first scenario, the fluid limitation is necessary in the case of hyponatremia secondary to SIADH, and the HS administration may be associated in this case (depending on the stage of neurological impairment). This approach is recommended to prevent iatrogenic problems, such as lung injury aggravation secondary to SARS-COV-2 infection and pulmonary edema. On the other hand, general guidelines establish the beginning of electrolyte replacement treatment in the case of hypovolemic hyponatremia secondary to GI fluid losses and decreased fluid intake (24). Therefore, personalized pathophysiological judgment is crucial in this pandemic since there are no official clinical guidelines for treating hyponatremia in COVID-19 patients.

As discussed above, the ideal concentrations of NaCl solutions in the different stages of COVID-19 and the associative consequences of NaCl unbalance and clinical relevance in COVID-19 patients remains unestablished. The ongoing RCTs on COVID-19 and HS approaches are summarized in Table 1. All trials are being applied to adults (≥18) or older adults among the RCTs. Three studies (NCT04465604, NCT04382131, and NCT05104372) are with the status of “recruiting” and one study had the status of completed (NCT04755972), whereas two others are in the level of “not yet recruiting” (Table 1).

**TABLE 1:** Summary of ongoing randomized controlled trials that included hypertonic saline approaches in the COVID-19 treatment.

Identifier	Therapy	Primary outcome measures	Last update posted	*n*	Prim. Purp.	Start date	Estim. study compl. date	Age	Status
NCT04465604	Surgical face mask sprayed with HS	Improvement of respiratory symptoms and respiratory signs	Mar 2021	50	T	Feb 2021	May 2022	≥18	Recruiting
NCT04382131	HS; nasal irrigation and gargling	Time to resolution of symptoms	Sep 2020	405	T	Jun 2020	Oct 2020	≥18	Recruiting
NCT04842721	HS; mouth Rinse Active Arm	Number of Participants with Negative COVID-19 PCR test results	May 2021	20	T	Jul 2021	Dec 2021	≥18	Notyet recruiting
NCT04341688	HS; gargle and nasal lavage	Intraoral viral load	Jul 2021	50	SC	Dec 2021	Jul 2022	≥18	Notyet recruiting
NCT04755972	HS; inhalation (active comparator)	Ventilator-associated pneumonia rate	Feb 2021	40	P	Jan 2021	Aug 2021	≥18	Completed
NCT05104372	HS; nasal irrigation and gargling	Time to resolution of symptoms	Nov 2021	405	T	May 2021	Nov 2021	≥18	Recruiting

*The studies were selected from the United States National Library of Medicine (assessed at ClinicalTrials.gov) by the descriptors “COVID-19 | NaCl Solution | Hypertonic saline”. Studies using only normal saline (until 0.9% sodium chloride) were excluded. n, estimated enrollment of participants; prim. purp., primary purpose; estim.compl. date, estimated completion date; T, treatment; SC, supportive care; P, prevention; HS, hypertonic saline; and PCR, polymerase chain reaction.*

### Hypertonic Saline and Neutrophils

Neutrophils’ altered responsiveness is likely to be a risk factor for severe COVID-19 considering increased mortality in the elderly, diabetic, and obese patients. These patients exhibit leukocyte dysfunction and chronic inflammation that predispose them to an excessive release of cytokines (70, 71). Impaired leukocyte function and reduced cell number are indicators of the progress from mild to severe clinical disease phases (72), and hyponatremia was recently associated with the high neutrophil count in SARS-CoV-2 patients (15).

The primary role of neutrophils in infections is the clearance of pathogens and debris through phagocytosis (73). The liberation of neutrophil-chemoattractant agents and the consequent recruitment of neutrophils is a vital host action against viral infection (74). Barnes et al. described an extensive lung infiltration of neutrophils in an autopsy specimen from a patient who succumbed to COVID-19 (75). Moreover, a high neutrophil-to-lymphocyte ratio indicates a poor prognosis for these patients (76).

Hypertonic solutions resuscitation has been reported as a potential strategy to decrease tissue damage and neutrophil activation in trauma patients (77), which mechanisms for neutrophil adhesion and sequestration vary with the inflammatory state. Chen et al. found that HS resuscitation has anti-inflammatory effects on panx1, CD39, CD73, and other ectonucleotidases. The authors also reported adenosine production induced by HS blocks neutrophil function through A2a receptors (78). Rizoli et al. investigated the effect of HS resuscitation on the progress of lung injury in a hemorrhagic shock model. They found suppression of LPS-stimulated activation and expression of CD11b. They demonstrated that CD11b integrin could be essential for neutrophil–endothelial interactions under the conditions studied (79). Reports also indicate that HS decreases lung injury by avoiding neutrophil adhesion to endothelium and suggests a mechanism for HS resuscitation. HS resuscitation decreases neutrophil margination by suppressing neutrophil L-selectin expression (80) and HS not only reduces post-shock mesenteric lymph release but also suppresses neutrophil priming by mesenteric lymph (81).

Hypertonic solutions may have clinical relevance by decreasing neutrophil-mediated intestinal damage. Tillinger et al. investigated HS treatment of neutrophils *in vitro*. They noted a dose-dependent effect involving decreased cell migration and the disruption of T84 monolayers compared with untreated control cells (82). Compared with physiological saline, Oreopoulos et al. found inhibition of ischemia/reperfusion-induced hepatic expression of ICAM-1 mRNA with HS from *in vivo* model of hepatic ischemia-reperfusion and *in vitro* model from the activated endothelial cell. The authors postulated hypertonicity minimizes neutrophil-mediated injury by regulating endothelial ICAM-1 expression (61).

**FIGURE 1:**
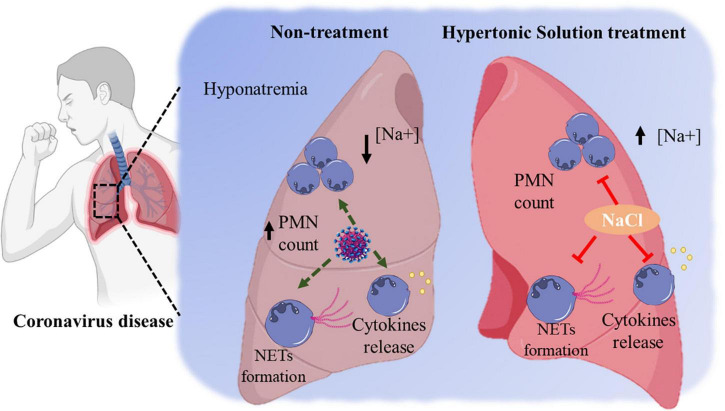
The possible beneficial effects of hypertonic saline solution decreasing neutrophils’ hyperresponsiveness in the coronavirus disease.

Hatanaka et al. reported that hypertonic NaCl solution strongly inhibits LPS-mediated cytokines released by neutrophils and mononuclear cells *in vitro* (83). These findings were corroborated by research showing that the blockage of surface integrins or selectin molecules expression by HS prevents the accumulation of neutrophils in the sites of inflammation (17). Besides, HS inhibits neutrophil’s function regarding the expression of adhesion molecules (84), reactive oxygen species (ROS) production (7), neutrophil migration (85), and exocytosis (86). However, the mechanism by which HS stimulates mucociliary clearance is not fully clarified yet. In addition, investigations on the effects of hypertonic NaCl solution on neutrophil death and ROS production should be encouraged. Figure 1 illustrates the possible beneficial effects of hypertonic saline solution decreasing neutrophils’ hyperresponsiveness in the coronavirus disease.

### Neutrophil Extracellular Traps: A Promising Path?

Neutrophil extracellular traps (NETs) play a role in immune defense, autoimmunity, and sepsis (87). NETs are constituted of antimicrobial agents and decondensed chromatin, including myeloperoxidase (MPO) and NE, which capture and kill bacteria, parasites, and fungi (88). Considering the high production of ROS and the cytokine storm, COVID-19 cases can be greatly worsened by the tissue-damaging actions of NETs (89). Conditions closely associated with NETosis are coagulopathy, severe tissue damage, and barrier dysfunction of the lungs (90). For an in-depth look at the subject, Borges et al. previously described the mechanisms related to NETs formation in the pathophysiology of COVID-19 (91).

Hypertonic solutions induces water to come out from the cell, activating various cellular processes (92), such as dehydration of neutrophils that mitigate their role to restrain ROS and sequential NETosis. Nadesalingam et al. described that HS usually used in therapies (509 mM or 3% saline) restrains NOX2-dependent NETosis promoted by phorbol-12-myristate-13-acetate (PMA) and LPS. They also reported that the suppressive action of HS on NETosis is in part controlled by restraining liberation of ROS, which is mainly exerted by an elevation in osmolarity (93).

Myeloperoxidase has been closely associated with hyper-inflammation tissue damage (94). Although the role of MPO in SARS-CoV-2 is still uncertain, NaCl on MPO may be clinically associated with less severe conditions. Delgado-Enciso et al. investigated ambulatory COVID-19 patients and therapy efficacy with nebulized and/or intravenous neutral electrolyzed saline (containing hypochlorous acid) associated with usual medical care versus routine medical care only. They found no adverse severe symptoms and showed an increased effect on SARS-CoV-2 clearance (95). The authors hypothesized that increased osmolarity influences oxidative processes related to the death and health deterioration of COVID-19 patients. However, the precise MPO mechanism still requires specific in-depth research to establish the effect of saline regarding the regulation of SARS-CoV-2 infectivity by MPO. This knowledge could assist the planning of novel salt approaches in intensive care unit-related inflammatory illnesses, such as COVID-19, in different stages of the disease.

## Final Considerations

Although the relationship between the electrolyte disturbances and COVID-19 infection is not fully clear yet, the electrolytic imbalance is often reported among COVID-19 patients, and hyponatremia is associated with a bad prognosis. HS therapies are simple, popular, economic, and feasible therapy to regulate leukocyte function with a robust anti-inflammatory effect in many inflammatory diseases, such as sepsis and cystic fibrosis. However, the potential anti-inflammatory action of HS *in vivo* requires more studies, the mechanisms by which HS stimulates mucociliary clearance are not fully clear and the cause-and-effect relationship of these events in patients with COVID-19 can only be confirmed by RCT. Therefore, there is a compelling need to investigate the effects of HS on neutrophil function and NETs formation in COVID-19 as promising targets for pharmacological treatment of the disease in the current pandemic scenario.

## Author Contributions

MG-F and LB developed the idea and wrote the manuscript. AD, EW, RC, and TP-C collected and prepared the study data. EH reviewed and edited the manuscript. All authors have read and agreed to the published version of the manuscript.

## Conflict of Interest

The authors declare that the research was conducted in the absence of any commercial or financial relationships that could be construed as a potential conflict of interest.

## Publisher’s Note

All claims expressed in this article are solely those of the authors and do not necessarily represent those of their affiliated organizations, or those of the publisher, the editors and the reviewers. Any product that may be evaluated in this article, or claim that may be made by its manufacturer, is not guaranteed or endorsed by the publisher.
