# Supplementary material for: Hypertonic Solution in Severe COVID-19 Patient: A Potential Adjuvant Therapy
Source: Front Med (Lausanne). 2022 Jun 21;9:917008. doi: 10.3389/fmed.2022.917008 (PMC9253300; doi:10.3389/fmed.2022.917008)
Supplement: Supplementary file 1 [file Table_1.DOC]

Supplementary Material

**Hypertonic Solution in Severe COVID-19 Patient: A Potential Adjuvant Therapy**

**Supplementary Material - Table S1.** Main clinical complications in COVID-19 patients.

| **Complications** | **Manifestations, Diseases, Syndromes** |
| --- | --- |
| Inflammatory | Exacerbated inflammatory response  Auto-antibody modulated manifestations  Toxic shock syndrome  Kawasaki disease |
| Respiratory | Pulmonary embolism  ARDS |
| Neurological | Acute hemorrhagic necrotizing encephalopathy  ADEM  Generalized myoclonus  Encephalopathy  Meningoencephalitis  PRES  Gullain-Barré syndrome (acute polyradiculoneuritis) |
| Cardiac | Acute cardiac injury  Arrhythmias  Shock  Cardiomyopathy |
| Secondary infections | Fungal/Bacterial coinfections |

Footnote: *Abbreviations* - acute respiratory distress syndrome, ARDS; acute disseminated encephalomyelitis, ADEM; posterior reversible encephalopathy syndrome, PRES.
